# Thermally Conductive Polyethylene/Expanded Graphite Composites as Heat Transfer Surface: Mechanical, Thermo-Physical and Surface Behavior

**DOI:** 10.3390/polym12122863

**Published:** 2020-11-30

**Authors:** Patrik Sobolčiak, Asma Abdulgader, Miroslav Mrlik, Anton Popelka, Ahmed A. Abdala, Abdelnasser A. Aboukhlewa, Mustapha Karkri, Hendrik Kiepfer, Hans-Jörg Bart, Igor Krupa

**Affiliations:** 1 Center for Advanced Materials, Qatar University, P.O. Box 2713 Doha, Qatar; patrik@qu.edu.qa (P.S.); asma.alkareem@qu.edu.qa (A.A.); anton.popelka@qu.edu.qa (A.P.); 2 Centre of Polymer Systems, University Institute, Tomas Bata University in Zlin, Trida T. Bati 5678, 76001 Zlin, Czech Republic; mrlik@utb.cz; 3 Chemical Engineering Program, Texas A&M University at Qatar, P.O. Box 23874 Doha, Qatar; ahmed.abdalla@qatar.tamu.edu; 4 Qatar Environment and Energy Research Institute, HBKU, P.O. Box 5825 Doha, Qatar; aaboukhlewa@hbku.edu.qa; 5 CERTES, Université Paris-Est Créteil Val de Marne, 94000 Paris, France; mustapha.karkri@u-pec.fr; 6 Chair of Separation Science and Technology, P.O. Box 3049 TU Kaiserslautern, 67653 Kaiserslautern, Germany; hendrik.kiepfer@mv.uni-kl.de (H.K.); bart@mv.uni-kl.de (H.-J.B.)

**Keywords:** multi-effect distillation, high density polyethylene, expanded graphite, polymeric composites, plasma treatment, scaling

## Abstract

Composites of high-density polyethylene (HDPE) and expanded graphite (EG) are prepared for heat exchangers in multi-effect distillation (MED) desalination. At 50 wt.% EG loading, the thermal conductivity of HDPE was increased by 372%. Moreover, the surface wettability of the HDPE/EG composite was enhanced by corona and RF plasma treatment as demonstrated by the increase in surface free energy from 28.5 mJ/m^2^ for untreated HDPE/EG to 55.5 and 54.5 mJ/m^2^ for HDPE/EG treated by corona and RF plasma, respectively. This enhanced surface wettability was retained over a long time with only a 9% and 18% decrease in RF and corona plasma-treated samples’ surface energy after two months. The viscoelastic moduli and the complex viscosity profiles indicated that EG content dictates the optimum processing technique. At loading below 30 wt.%, the extrusion process is preferred, while above 30 wt.% loading, injection molding is preferred. The plasma treatment also improved the HDPE/EG composite overall heat transfer coefficient with an overall heat transfer coefficient of the composite reaching about 98% that of stainless steel. Moreover, the plasma-treated composite exhibited superior resistance to crystallization fouling in both CaSO_4_ solution and artificial seawater compared to untreated composites and stainless-steel surfaces.

## 1. Introduction

Seawater thermal desalination provides over 90% of the water demand for Qatar and other Gulf Countries. Multi-effect distillation (MED) is the most energy-efficient thermal desalination process. However, due to the high salinity of Arabian Gulf water, metal heat exchangers used in MED desalination units suffer from severe corrosion, fouling, and scaling problems. The current practice to cope with these issues involves using expensive metal alloys and frequent cleaning and scale removal, leading to increased capital and operational costs. 

Compared to metals, polymers are less susceptible to corrosion, fouling, and scale formation, but they have very low thermal conductivity and are mechanically weaker. However, polymer composites with thermally conductive fillers can enhance the polymer thermal conductivity and improve its mechanical properties [1,2,3,4,5]. In addition, the low polymer density and ease of processing are additional benefits offered by polymers, which contribute to the reduction of the investment cost [2]. 

The use of innovative heat exchangers of corrosion-resistant polymer and polymer-nanocomposites may provide a less costly alternative to heat exchangers made of expensive metallic materials (titanium, Cu-Ni alloys, stainless steel, and Al-brass). Nevertheless, the majority of the current commercial polymer heat exchangers are designed as spirals or tube bundles, and to compensate for their low thermal conductivity, the heat transfer surfaces are fabricated as very thin films [3]. An overview of the applications of polymers in heat exchangers is given published by Reay [4], Zaheed and Jachuck [5], and Cevallos et al. [6]. 

Scheffler and Leao [7] reported the fabrication of polymer heat exchanger, and Christmann et al. [8] used polyetheretherketone (PEEK) film as a heat transfer surface and achieved overall heat transfer coefficients of up to 1570 ± 181 W/m·K depending on the operating conditions [8]. Simulation and experimental work indicated that a polymer (PEEK) film with a thickness of 25 μm and appropriate spacer geometry provides sufficient mechanical stability for the heat exchanger under MED conditions [8].

The notable developments in the last decade and primary potential applications for polymer heat exchangers, including solar water heaters, heat recovery systems, and seawater heat exchangers, in particular, for the desalination industry are discussed elsewhere [6,9]. The application of polymeric hollow fiber heat exchangers for the thermal desalination process was recently reported, and an overall heat transfer coefficient of ~2000 W/m^2^·K for brine-water systems was obtained using polypropylene hollow fibers [10].

Although these reported polymer heat exchangers achieved a high overall heat transfer coefficient, the extension of their application to large scale processes is limited due to their very low thickness, e.g., 25 μm for PEEK, which prevents their use in long tubes due to the intrinsic low mechanical strength of polyolefin polymers. Therefore, there is a need to enhance these polymers’ thermal conductivity to allow for the use of reasonable thickness (>500 μm), which can be fabricated by the conventional extrusion process for tubes and compression molding for plates. Moreover, because the heat transfer coefficient is a function of the wall thermal conductivity and thickness, wettability, and fouling factor, it is necessary to enhance the mechanical properties, control wettability, and reduce susceptibility to crystallization fouling in addition to increasing the thermal conductivity of the polymer. 

Low-temperature plasma treatment represents an effective route for improving the wettability of the HDPE surface [11,12]. Generally, three types of radicals can be created on the HDPE surface, namely alkyl, allyl radicals, and dangling bond sites with different stabilities in the first stage [13]. These functional radicals can cause crosslinking, creating double bonds in the polymeric layer or the formation of oxidized functional groups on the surface responsible for a wettability increase [14,15]. The presence of a filler in the polymeric matrix can positively affect wettability improvement after plasma treatment. Plasma treatment of linear low-density polyethylene/graphene nanoplatelets composite led to a significant wettability improvement [16].

Polymers have intrinsically low thermal conductivity (0.2–0.4 W/m·K). Hence, enhancing thermal conductivity is required for many applications, such as the thermal management of electronic equipment, LEDs, and heat transfer applications. Increasing the thermal conductivity of a polymer is achieved via the incorporation of thermally conductive nanofillers, such as carbon materials, metals, and ceramics. In this section, we summarize the reports of polymer composites with high thermal conductivity. However, we exclude composites filled with parallel oriented continuous fibers due to their poor conductivity in the orthogonal (heat flow) direction, their high cost associated with the very high fiber loading, and their challenging processing. 

Bujard [17] developed composites based on epoxy resin filled with small flake-like boron nitride crystals. A composite with a 31 vol.% filler content achieved thermal conductivity of 2.3 W/m·K. In another article, Bujard et al. [18] reported a thermal conductivity of 4.5 W/m·K for bisphenol-F epoxy resin filled with 80 vol.% alumina flakes and 4.25 W/m. K for epoxy resin filled with 62 vol.% alumina nitride [19]. 

An extremely high thermal conductivity of 32.5 W/m·K was reported by Ishida et al. [20] for polybenzooxazine composite with 78.5 vol.% boron nitride. This very high thermal conductivity was attributed to the outstanding properties of the polybenzoxine matrix and the boron nitride filler. The bisphenol-A-methylamine based polybenzoxazine possesses a low viscosity, which aids filler wetting and dispersion. The 225-μm average size filler particles form large aggregates of boron nitride flake-like crystals. Moreover, this filler has a bimodal size distribution, which assists in increasing the particle packing density. The filler-matrix system provides a highly thermally conductive composite due to the capability to form conductive networks with low thermal resistance. The authors also mentioned the significant anisotropy of this system. However, they did not report the thermal conductivity values in the different directions.

SGL Carbon Company reported very high thermal conductivity for composites based on thermoplastic polymers with expanded EG with a 200–500 μm particle size (SGL Carbon datasheet). When polypropylene was filled with 80 wt.%. The thermal conductivity in the orthogonal direction was 23 W/m·K and the in-plane conductivity was 60 W/m·K. Similarly, polyamide 6,6 filled with 60 wt.% EG achieved thermal conductivity in the orthogonal direction of 6 W/m·K and in plane conductivity of 26 W/m·K.

High thermal conductivities for polymer composites with nanofillers such as graphene nanoplatelets have been recently reported at relatively low filler loading than the above composites. Shtein et al. [21] reported a thermal conductivity of 12.5 W/m·K for an epoxy-graphene nanoplatelet (GnP) composite at GnP loading of 25 vol.% due to their high aspect ratio, i.e., 1255. 

Herein, polymeric composites based on HDPE and EG suitable for MED are proposed. Corona and RF plasma were used to improve the wettability of HDPE/EG. The scaling experiments were conducted with two experimental systems, namely CaSO_4_ and artificial seawater. For both test systems, the polymer composites’ plasma treatment leads to a considerable reduction of the fouling tendency. 

Proposed polymeric composites represent a promising path towards the replacement of metallic materials in MED. Plasma treatment of polymer composites significantly improved fouling, which is one of the major challenges of MED technologies.

## 2. Materials and Methods

### 2.1. Materials

High density polyethylene (HDPE, Q3802, Q-Chem, Doha, Qatar) and expanded graphite (EG, GFG200, SGL Carbon, Wiesbaden, Germany) having an average size of 200 μm were used for the composite preparation. CaCl_2_ 2 H_2_O (Sigma Aldrich^®^, St. Louis, MO, USA, purity ≥ 99 %) and Na_2_SO_4_ (Sigma Aldrich^®^, purity 99.5%), formamide (Sigma Aldrich^®^, purity ≥ 99.8%), and ethylene glycol (Sigma Aldrich^®^, purity ≥ 99%) were used. Ultrapure water was obtained using an Ultrapure Water System NW Series (Heal Force Bio-Meditech Holdings, Ltd., Shanghai China).

### 2.2. Composite Preparation

HDPE-EG composites with EG in various loadings (5, 10, 15, 20, 25, 30, 40, and 50 wt.%) was melt mixed using a batch mixer Brabender Instrument (Plastograph EX, Brabender, Pfullingen, Germany) at 180 °C for 10 min. Subsequently, the blends were hot-pressed (Carver, Wabash, IN, USA) at the pressure of 3 tons at 160 °C for 3 min. Plasma treatment, wettability study, heat transfer, and scaling experiment was done with the composite containing 50 wt.% of EG.

### 2.3. SEM Analysis

The composite’s fractured surface was inspected by a Nova Nano SEM 450 scanning electron microscope (SEM, Osaka, Japan) operating at 20 kV. Brittle fracture of the specimen was achieved by immersing the specimen in liquid nitrogen for 30 s and subsequent breakage of the specimens.

### 2.4. Plasma Treatment 

Prior to the plasma treatment, the HDPE and HDPE/EG films were washed by acetone to eliminate any dust or possible contaminations from the production process that could affect the surface properties and then air-dried at room temperature for 20 min. Small strips (7 cm × 1.5 cm) were cut out and directly used for surface treatment and various analyses. 

The low-temperature plasma treatment was carried out using a radiofrequency (RF) and corona plasma system. The vacuum pressure system generating RF plasma was Venus75-HF (Plasma Etch Inc., Carson, CA, USA). During the plasma treatment, the ions and electrons in this system were generated by an RF power supply operating at a typical frequency of 13.56 MHz. The process parameters, such as treatment time, were adjusted to optimize the plasma treatment process to obtain the maximum hydrophilicity (wettability). The chamber was evacuated to a vacuum level of 0.2 Torr, and the treatment time applied varied from 10 s up to 120 s at 80 W of nominal power. Corona treatment in the air was done under atmospheric pressure. The nominal power was fixed to 300 W with a time increase from 1 to 10 s to study the time effect on the surface properties enhancements. The distance between the plasma generating electrodes and the film surface was fixed to 1 mm to ensure homogeneous plasma discharge.

### 2.5. Rheological Investigation

The HDPE-based composites’ melt rheological properties were investigated at 140, 160, and 180 °C using a Physica rotational rheometer (MCR502, Anton Paar, Graz, Austria) equipped with a Peltier heating/cooling fixture and parallel-plate geometry (PP25). A discoid sample with 1-mm thickness was placed between the parallel plates and subjected to oscillatory shear. The possible sample slippage was efficiently reduced by using a constant normal force of 0.3 N. The linear viscoelasticity region (LVR) was determined by amplitude-sweeps in the strain range of 10^−2^ to 10% at a frequency of 1 Hz. Furthermore, a frequency-sweep from 5 × 10^−2^ to 50 Hz was performed with a strain that ensures the samples stayed in the (LVR). 

### 2.6. Mechanical Analysis

Tensile measurement was carried out at room temperature according to ASTM D638 using dog-bone specimens cut from 1 mm hot-pressed slabs using a Lloyd Instruments LF Plus Digital Materials Tester (Ametek San Diego, CA, USA) equipped with 1-kN load cell with a strain rate of 10 mm/min. Each sample was measured using five specimens, and the average value and standard deviation are reported.

### 2.7. Thermal-Transport Measurement

The thermal conductivity (λ) and thermal diffusivity (α) of the pure HDPE and its composites are measured using the periodic temperature ramp method [22] that allows the simultaneous measurement of the thermal conductivity and thermal diffusivity. The parallelepiped-shaped sample (45 mm × 45 mm × 5 mm) is fixed between two metallic plates, and a conductive grease (λg = 1 W/m·K) is used to ensure good thermal exchange between the sample and the two metallic plates. 

### 2.8. Differential Scanning Calorimetry (DSC)

The DSC measurements were performed using a Perkin Elmer, DSC 8500 (Perkin Elmer, Greenville, SC, USA) with 5–8 mg of sample heated/cooled under a nitrogen environment at a rate of 5 K/min. The specific heat capacity was measured using a three-step method, which included baseline, sapphire (as a standard), and sample measurements. The specimen was cooled to 15 °C at a rate of 5 °C/min, then held at this temperature for 2 min, subsequently heated to 30 °C, and finally kept at 30 °C for 2 min. All experiments were repeated at least three times.

### 2.9. Wettability Investigation 

The change in the composite’ s hydrophilicity after plasma treatment was assessed by measuring the static contact angle using an optical contact angle measuring system (OCA35, DataPhysics, Filderstadt, Germany) equipped with a CCD camera. Three testing liquids (water, formamide, and ethylene glycol) were used to evaluate HDPE and HDPE/EG composite wettability. A droplet of ~3 μL from the testing liquid was dispensed on the sample, and the contact angle was calculated after 3 s to allow for thermodynamic equilibrium between the liquid and the sample surface. Five independent measurements were taken from different positions of each sample, and the average contact angle of each liquid is reported. The surface free energy has been evaluated using the Owens-Wendt-Rabel-Kaelble method. The durability and stability tests are essential for understanding the aging effects that occurred in plasma-treated surfaces. The aging was analyzed for HDPE and HDPE/EG treated by RF and corona plasma system and stored in air at RT and relative humidity of 45% for two months of aging. 

### 2.10. Heat Transfer and Scaling Measurement

**Scheme 1:**
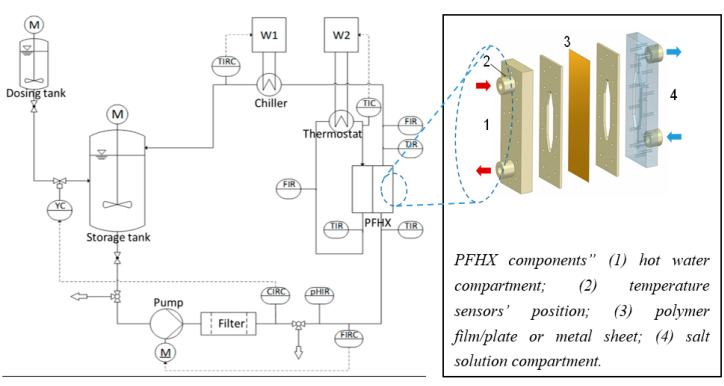
(**left**) Process flowchart of the experimental set-up and (**right**) detailed view of the PFHX.

The heat transfer and scaling formation measurements were performed using a home-made setup, schematically depicted in Scheme 1, and described in more detail elsewhere [23]. The test cell (4) is a mini polymer film heat transfer cell (PFHX) operating at counter-current flow with a heat transfer area of about 32 cm^2^. The overall heat transfer coefficient and the kinetics and quantity of crystallization fouling were studied using this setup.

A polymer (HDPE or HDPE/EG composite) or a metal (stainless steel) plate separates the solution from the heating side (hot water) in the test section. The heating side of the PFHX is manufactured of POM to ensure adiabatic conditions, and the solution side is made of PMMA to allow for visual inspection of the polymer sheet. Temperature sensors (Pt 100 1/3 DIN) are directly inserted in the bulk flow at the inlet and the outlet of the test PFHX (see arrows in Scheme 1), where the temperature distribution and the flow profile are homogeneous. 

The scaling experiments were conducted to evaluate the polymer composites’ fouling behavior in aqueous solutions containing salts with inverse solubility. The hot-side fluid was either artificial seawater (45 g salt per kg) with composition, according to Kester et al. [24] or 25 mmol/L CaSO_4_-solution prepared by mixing CaCl_2_ × 2 H_2_O with Na_2_SO_4_ into pre-heated deionized water to prepare.

During the experiments, the inlet and outlet temperatures and the volumetric flow rates were controlled and recorded. Once a steady-state condition had been achieved, data were recorded for 15 min and then averaged to determine the overall heat transfer coefficient.

## 3. Results and Discussions

**Figure 1:**
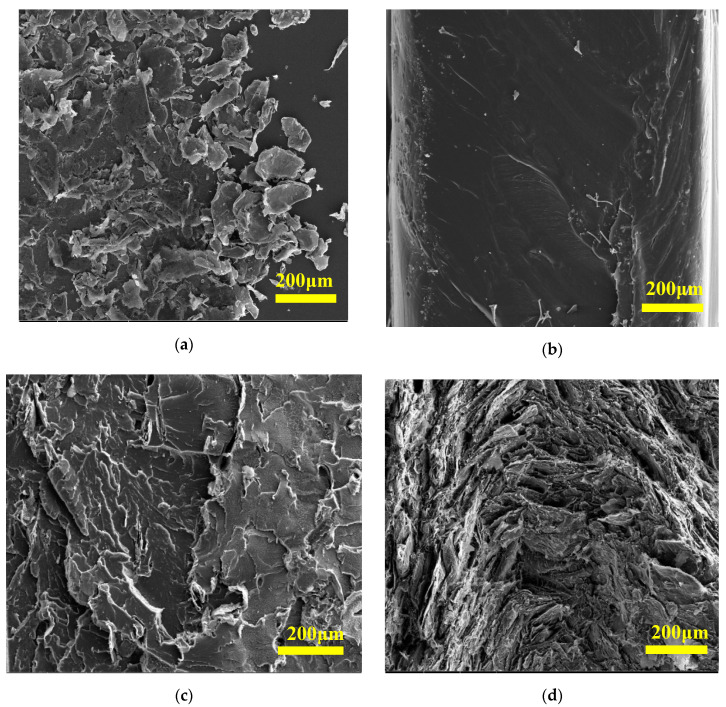
SEM images of (**a**) EG filler, (**b**) neat HDPE, (**c**) HDPE-10 wt.% of EG and (**d**) HDPE- 50 wt.% of EG.

The morphology of prepared composites has been studied using SEM analysis by investigating the cross-section of composites. Figure 1a shows the SEM image of expanded graphite. The size of EG is around 200 μm, consistent with that reported by the manufacturer. The fracture surface of the neat HDPE structure appears as a relatively flat surface without any defects, as shown in Figure 1b. The morphology of the HDPE composite containing 10 wt.% of EG (Figure 1c) reveals no evidence of agglomeration of EG. Similarly, the HDPE composite containing 50 wt.% of EG (Figure 1d) exhibited good homogeneity of the EG distribution, which is the mandatory requirement for improving thermal and other composite properties.

### 3.1. Thermo-Physical Properties

**Figure 2:**
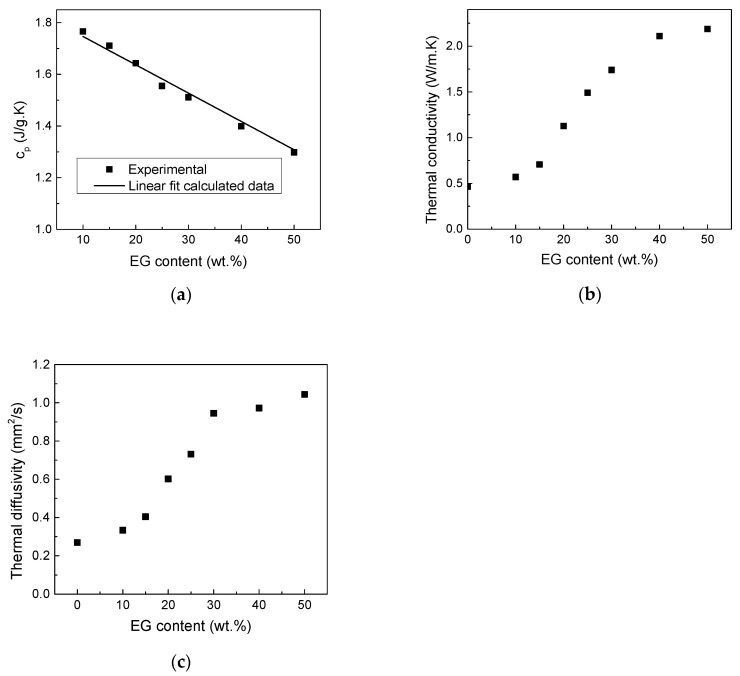
(**a**) experimental and calculated $c_{p}$ of HDPE, and EG composites by weight, (**b**) thermal conductivity of HDPE and EG composites and (**c**) thermal diffusivity of HDPE and EG composites.

The specific heat capacity ($c_{p}$) of pure HDPE and EG and their composites was measured by DSC and the results are shown in Figure 2a. The specific heat capacity of the composite samples decreases linearly with EG loading following an additive mixture rule (Equation (1)) represented by the solid line in Figure 2a, confirming the dense nature of the composite and the absence of any voids or air bubbles.

(1) $$c_{p,c}=c_{p,p}\left(1-\frac{w_{f}}{100}\right)+c_{p,f\;}\frac{w_{f}}{100}$$

where $c_{p,p}$ is $c_{p}$ of neat HDPE (1.84 Jg^−1^ C^−1^) and $c_{p,f\;}$ is $c_{P}$ of EG (0.76 Jg^−1^ C^−1^) 

Moreover, the thermal transport parameters, λ and α, provided in Figure 2b,c revealing that the addition of EG significantly improves λ and α of the HDPE-EG composites, particularly at higher EG concentrations as generally expected. In both cases, a non-linear increase in λ with increasing EG mass fraction was observed. This result is attributed to the high λ of EG and its shape [25]. The highest increase in λ is 372% for composites filled with 50 wt.% of EG.

### 3.2. Rheological Properties

**Figure 3:**
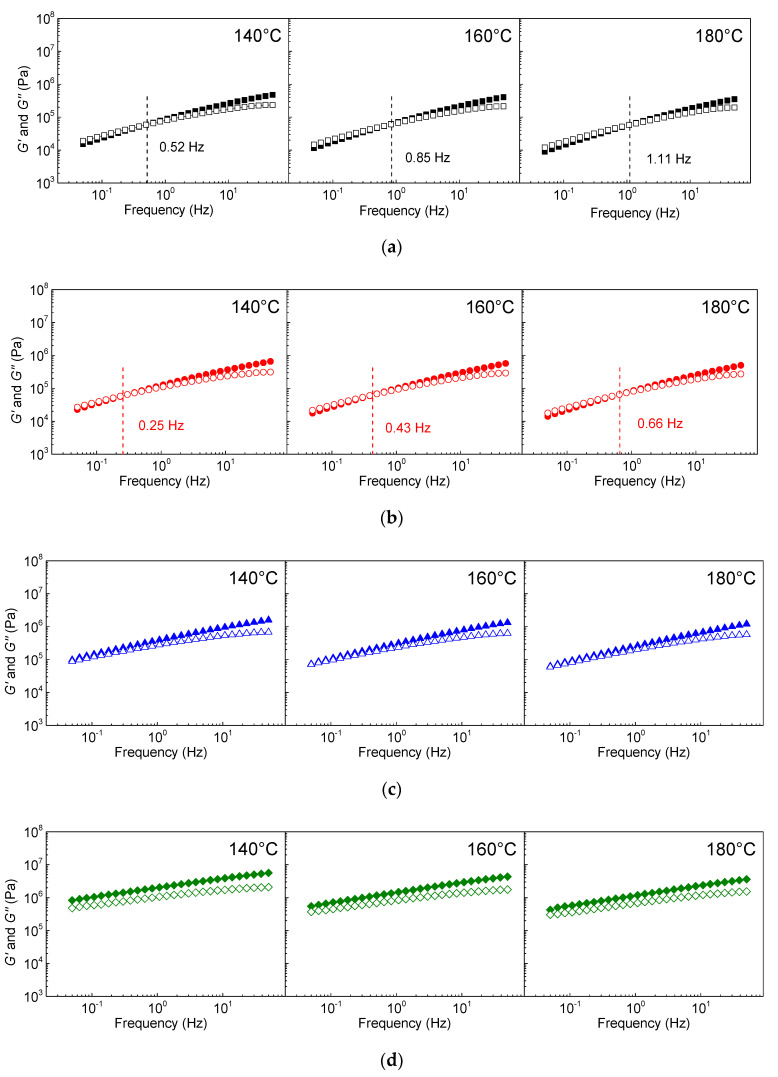

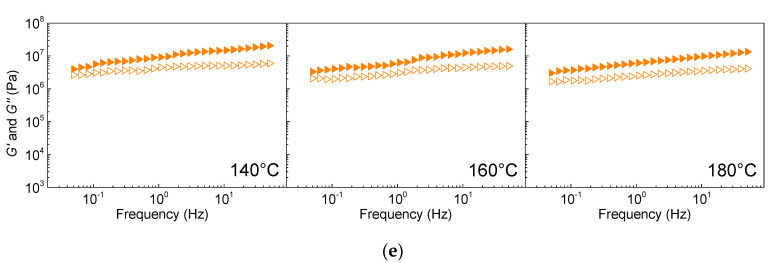
Dependence of storage, G’ (full symbols), and loss modulus, G’’ (empty symbols), on the frequency for (**a**) neat HDPE, (**b**) HDPE/EG 90/10, (**c**) HDPE/EG 70/30, (**d**) HDPE/EG 50/50 and (**e**) HDPE/EG 30/70 for various temperatures.

To investigate the HDPE-EG composites’ processability, the elastic and viscous moduli at 140, 160, and 180 °C are measured in the frequency range 10^−2^ and 1 rad/s, and the results are shown in Figure 3. The neat HDPE exhibits typical Maxwellian behavior at all investigated temperatures with cross-over frequency at 0.5, 0.8, and 1.1 Hz at 140, 160, and 180 °C, respectively, marking liquid-like to solid-like transition due to the pseudoplastic behavior of HDPE [26]. However, with the addition of 10 wt.% of EG, both G’ and G” increase, and the cross-over frequency shifts to a lower frequency, relative to pure HDPE, indicating enhanced viscoelastic behavior of the polymer melt consistent with previously reported results [27]. 

As the EG’s content increases to 30 wt.%, the cross-over frequency shifts to a much lower frequency and becomes undetectable in the studied frequency range (0.05–100 Hz). As the EG loading increases further, G’ becomes much higher than G” over the entire frequency range, implying the enhanced elastic component due to the formation of a percolated network by the EG sheets. The percolated network formation is also implied by the decrease in the slope of the G’ and G” vs. frequency with increasing EG loading. 

The enhancement in the polymer melt’s viscoelastic behavior at high EG loading requires the composites’ processing with EG loading of 50 or 70 wt.% at an elevated temperature of 180 °C [28]. Furthermore, the addition of the EG significantly stiffens the polymer melt [29], and further processing might be possible for HDPE/EG 50/50 (*w*/*w*) or 30/70 (*w*/*w*) at elevated temperatures above 180 °C but only using injection molding techniques of highly-filled systems [30].

**Figure 4:**
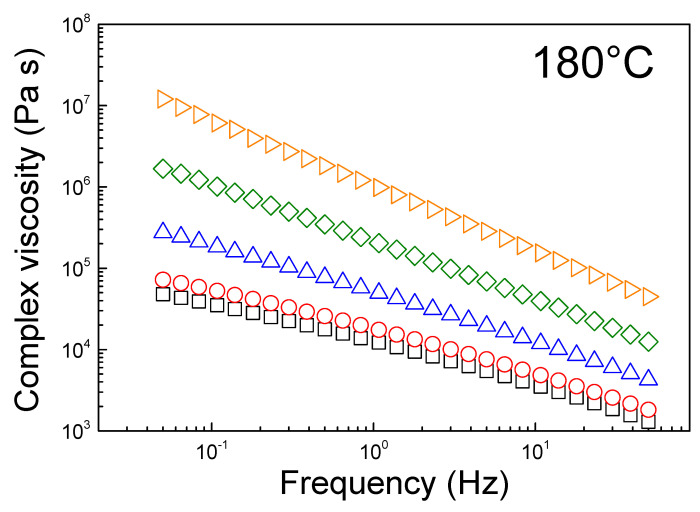
Dependence of complex viscosity on the frequency for various neat HDPE and various HDPE/EG composites at 180 °C, where ☐—neat HDPE, ◯—90/10, △—70/30, ◇—50/50, ◁—30/70.

The challenging processing of the composites with high EG loading is implied by the very complex viscosity at 180 °C, as shown in Figure 4. The complex viscosity versus frequency is strongly dependent on the EG loading, and it increases by over two orders of magnitudes as the EG loading is increased to 70 wt.%. Both neat HDPE and HDPE/EG 90/10 (*w*/*w*), whose zero-shear viscosities are <10^5^ Pa.s, can thus be processed via extrusion, while the complex viscosity of HDPE/EG 70/30 (*w*/*w*) has significantly increased by half an order of magnitude. A further increase of EG concentration to 50/50 (*w*/*w*) and 30/70 (*w*/*w*) leads to a significant increase in the low-frequency viscosity to exceed 106 Pa.s. Moreover, the absence of a “Newtonian” plateau at low frequency and the strong shear thinning profile for all samples indicate the high shear dependent melt viscosity due to the strong entanglement of the high molecular weight HDPE chains. Therefore, the highly-filled HDPE composites with EG loading >50 wt.% require to be fabricated using injection molding techniques at or above 200 °C [30].

### 3.3. Mechanical Properties

**Figure 5:**
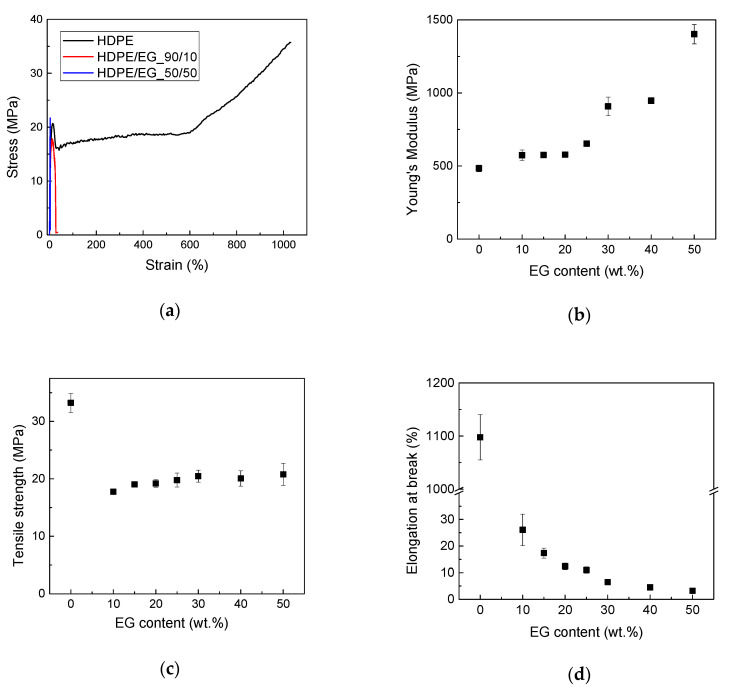
(**a**) stress-strain curve of HDPE and composites contain 10 and 50 wt.% of EG, (**b**) Young’s modulus HDPE and composites, (**c**) Tensile strength HDPE and composites, and (**d**) elongation at break HDPE and composites.

The mechanical properties of HDPE and HDPE-EG composites were measured via tensile testing. As expected, Young’s modulus increases with EG content and reaches 1440 MPa at 50 wt. % EG as shown in Figure 5. This increase in stiffness is attributed to the stiffening of the polymer chains caused by the filler’s confinement of the HDPE chains. Although the increase in modulus could also result from increased crystallinity, this is unlikely as EG’s addition did not improve the polymer crystallinity. Moreover, the enhanced modulus suggests an increase in the hardness and high deflection temperature (HDT) of the composite.

On the other hand, the ultimate stress (stress at break) of semicrystalline polymer composites has a more complex dependence on the filler concentration, where the tensile strength is expected to increase by the reinforcing effect of the filler. In contrast, the strength could decrease due to the filler’s negative impact by restricting the orientational changes of the semicrystalline polymers at high deformation, as seen in Figure 5a. At low filler contents, the deformation is low enough to prevent the orientation, but the filler presence’s reinforcing effect is marginal. Therefore, an initial decrease in tensile strength has been observed. With the increase in the filler content, the reinforcing effect is more pronounced, while a further decrease in deformation has no additional effect on orientation. Another important factor that dictates the change in the tensile strength is the polymer-filler interface’s strength and ability to transfer the load from the polymer to the filler. The weak interface between the non-polar HDPE and the EG filler is expected to reduce the load transfer and limit the filler’s strengthening effect.

The elongation at break decreased with the addition of the filler, consistent with what is commonly experienced with many polymeric composites as the elongation at break decreases from over 1000% for neat HDPE to 26% and 3% for HDPE-EG composites containing 10 and 50 wt.% EG, respectively, as shown in Figure 5d. 

### 3.4. Wettability Analysis

**Figure 6:**
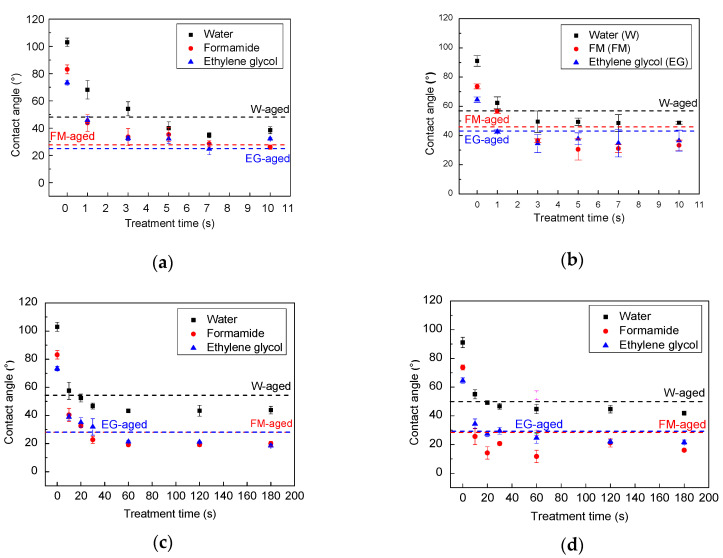
Contact angle of: (**a**)—corona treated HDPE, (**b**)—corona treated HDPE/EG, (**c**)—RF treated HDPE, and (**d**)—RF treated HDPE/EG. Dashed lines represent plasma treated samples for 7 s and aged 2 months.

**Figure 7:**
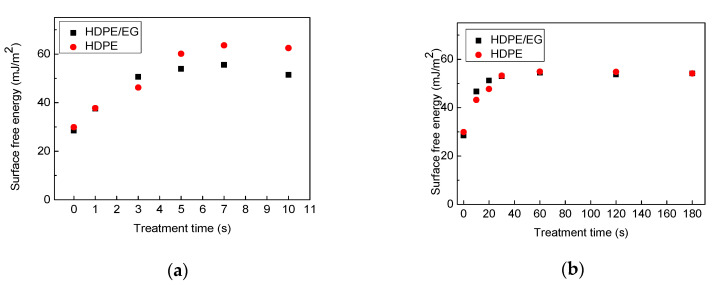
Effect of treatment time on the surface free energy of HDPE and HDPE/EG treated by (**a**) corona and (**b**) RF plasma.

The changes in the wettability of the HDPE and HDPE/EG composites induced by plasma treatment are evaluated by measuring the contact angle of three testing liquids. First, the plasma treatment parameters (plasma type, nominal power, and treatment time) were optimized. Moreover, by measuring the contact angle of liquids with different surface tensions (water, ethylene glycol, and formamide) (Figure 6), the surface free energy has been determined, and the results are presented in Figure 7.

The HDPE is a hydrophobic polymer, and therefore it exhibited a relatively high contact angle of 103.0°, 83.1°, and 73.3° for water, formamide, and ethylene glycol, respectively (see Figure 6a). Moreover, the addition of 50 wt.% EG to HDPE led to slightly improved wettability due to the roughness increase, and therefore, the contact angles decreased to 91.1°, 73.7°, and 64.5° for water, formamide, and ethylene glycol, respectively (see Figure 6b).

After plasma treatment, the surface of HDPE and HDPE-EG composite became hydrophilic as marked by the remarkable decrease in the contact angles even using short treatment times (1 s for corona and 10 s for RF plasma treatment) due to the formation of new polar functional groups and etching processes on the surface during the plasma treatment process. With longer treatment times, e.g., 7 s for corona, and 60 s for RF plasma system, the maximum improvement in the surface wettability (hydrophilicity) was reached with no significant changes after using longer treatment times. The impact of corona and RF plasma treatment on the wettability of HDPE and HDPE/EG is shown in Figure 7.

Consistent with the contact angle results, the surface free energy for HDPE and HDPE/EG has significantly increased, from 29.9 mJ/m^2^ and 28.5 mJ/m^2^ to 63.6 mJ/m^2^ and 55.5 mJ/m^2^ after 7 s of corona treatment, respectively, and to 54.9 mJ/m^2^ and 54.5 mJ/m^2^ after 60 s of RF plasma treatment, respectively. The increase in the free energy validates HDPE and HDPE/EG surfaces’ functionalization, which confirms that plasma treatment results in better wettability. This effect can be explained in terms of the polarity of the solid-liquid interface. More polar groups (functionalization processes) result in increased molecular forces, consequently enhancing the interactions between the two surfaces (polymer-testing liquid), which are in contact, hence the hydrophilicity of the surface increases. 

The impact of corona and RF plasma treatment on the wettability of HDPE and HDPE/EG is shown in Figure 7.

Compared with the HDPE, HDPE/EG showed more hydrophilic character than HDPE at shorter plasma treatment times (3 s for corona, 20 s for RF) since the increase in the surface free energy using air plasma was more remarkable for HDPE/EG than HDPE samples. This phenomenon can be associated with incorporating polar functional groups into the EG nanoparticles after plasma treatment, resulting in improved overall hydrophilic properties of HDPE/EG [31]. However, HDPE’s corona plasma treatment led to a more significant increase in the wettability compared with HDPE/EG using longer treatment times of 5 s. The plasma effect on the polymeric matrix was probably more dominant than on the filler. On the contrary, a plasma treatment time of 40 s and more using the RF system had an almost identical effect on improved wettability of both HDPE and HDPE/EG. 

An aging study has been carried out for an extensive-time period (two months) after the plasma treatment to study the long-term stability of the wettability of the HDPE and HDPE/EG surface. The aging phenomenon mainly depends on the storage condition, material properties, and treatment type. Figure 6 also presents the water contact angle’s evolution with aging time for HDPE and HDPE/EG composite treated with RF and corona plasma system and stored in an air environment. Some functional groups are subjected to aging with storing time, often called “hydrophobic recovery,” while the roughness remains the same, and only partial hydrophobic recovery will occur [32]. As can be seen, the plasma treatment led to a slight increase in the contact angles and a decrease in the surface free energy for both HDPE and HDPE/EG aged samples modified by corona (7 s treatment time) and RF plasma (60 s treatment time). The surface underwent an aging process was analyzed after two months, while the samples exhibited only a slight deterioration of wettability. We suppose that this phenomenon was caused by the rotated and diffusion of oxygen-containing groups from the modified polymer surface into the bulk during the aging process. This slight deterioration of wettability was more pronounced for the HDPE and HDPE/EG samples treated by the corona plasma system. The surface free energy of RF plasma-treated HDPE/EG samples decreased only by 9% after aging time since the EG particles could act as a barrier for the oxygenated groups’ motion compared with a 14% decrease for the neat HDPE sample. Slightly less difference between the hydrophobic recovery of HDPE/EG and HDPE was observed using corona plasma treatment, while the surface free energy decreased by about 18% and 20% for HDPE/EG and HDPE, respectively.

### 3.5. Heat Transfer Measurements

Table 1 provides the overall heat transfer coefficients measured using the setup detailed in the experimental section under various conditions. Plasma treatment leads to a 6–24% increase in the overall heat transfer coefficient. Moreover, the overall heat transfer coefficient of the plasma-treated composite sheet under laminar flow conditions in the cold water side was about 98% of the overall heat transfer coefficient of stainless steel regardless of the low thickness of the stainless steel sheet (1 mm) compared to the thickness of the composite sheet (2 mm). This remarkable result indicates that the composite HDPE sheet with thermal conductivity of ~2.8 W/m·K can be useful as a heat transfer surface. 

Moreover, the overall heat transfer coefficient for neat HDPE was only 25% of the HDPE-EG composite coefficient with 50% EG, confirming thermally enhanced polymer composites’ potential in heat exchanger applications.

### 3.6. Fouling Resistance Analysis

Scaling experiments were conducted with two scale-forming fluids, i.e., CaSO_4_ and artificial seawater, and the heat transfer resistance due to fouling (fouling resistance) of untreated and plasma-treated HDPE-EG (50/50) is measured and compared to the fouling resistance of stainless steel. 

**Figure 8:**
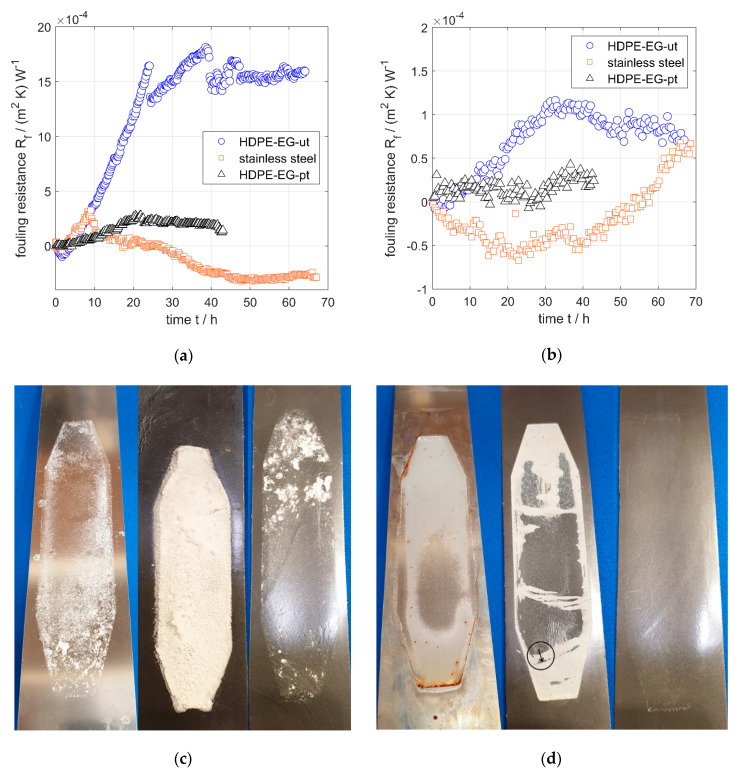
Measured fouling resistance due to crystallization fouling of (**a**) CaSO_4_, (**b**) mixed salts from seawater on the investigated heat transfer films. w = 45 g/kg; $\dot{q}$ = 35 kW/m²; Tb = 30 °C; Resol = 3100, (**c**) scaling layer of CaSO_4_ and (**d**) scaling layer caused by artificial seawater on stainless steel 1.4301 (left), HDPE-EG-ut (middle), HDPE-EG-pt (right).

A fouling behavior of the three test samples (HDPE-EG-ut, HDEP-EG-pt, and stainless steel) in 4.5 g/kg CaSO_4_ solution was analyzed. The fouling of untreated HDPE-EG (50/50) was increased rapidly during the first 25 h then reached a plateau, while the stainless surface was experienced a similar initial increase in fouling in the early times (t < 10 h), but followed by a sharp decrease in fouling possibly due to detachment of the fouling layer formed in the early experimental stage. On the other hand, the plasma-treated HDPE-EG samples have a much lower rate of fouling over the entire testing time of 40 h, as shown in Figure 8a. This behavior was confirmed by the three surfaces’ optical imaging at the end of the scaling experiments, as shown in Figure 8c that clearly shows the very fouled HDPE-EG surface and the almost clean HDPE-EG-pt surface. 

In the experiments with artificial seawater, untreated HDPE-EG exhibited increasing fouling resistance up to 30 h, followed by a slight decrease, as shown in Figure 8b. In contrast, the plasma-treated sample experienced no fouling with artificial seawater up to 40 h, which was also confirmed by the optical image provided in Figure 8d that reveal. The HDPE-EG-pt composite is free of any fouling layer, unlike HDPE-EG-ut, which contains a visible layer on the surface. On the other hand, the stainless-steel specimen shows a long induction phase, but after about 40 h, there is a substantial increase in fouling resistance, partly caused by corrosion, which is avoided by using polymers.

**Figure 9:**
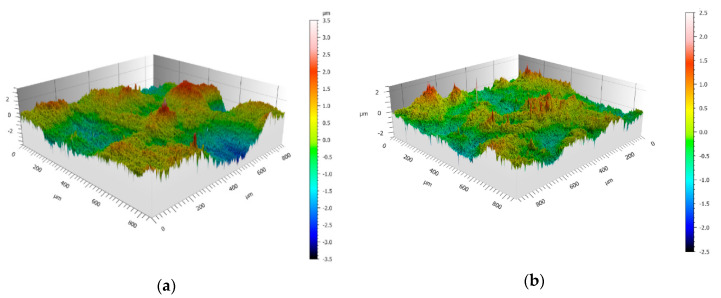
AFM surface profile of HDPE-EG50 composite (**a**) before and (**b**) after plasma treatment.

The excellent resistance to fouling of the plasma-treated HDPE-EG is attributed to the resulted modified surface properties. Moreover, the surface roughness and topographic structures of the investigated materials were analyzed using confocal microscopy, and the 3D topographic images for the untreated and plasma-treated samples are illustrated in Figure 9, and the roughness parameters are summarized in Table 2. 

The roughness analysis and the 3D topographic images demonstrate that the plasma-treated polymer has a significantly reduced surface roughness compared to the untreated sample. On the other hand, stainless steel has the lowest roughness but shows no significant difference in fouling susceptibility compared to the plasma-treated composite. The positive effect of plasma treatment is attributed to the associated change in the free surface energy and surface chemistry. The plasma treatment leads to a significant increase in the free surface energy, leading to a reduced fouling tendency. However, stainless steel’s surface free energy is about 33 mN/m [33], which lies between that of the plasma-treated and untreated polymer composite. It is worth noting that there is no simple correlation between surface energy or roughness and fouling behavior has already been shown by various authors [33,34], which is confirmed by the results shown here. Nevertheless, the plasma treatment of the polymer composite leads to a significantly reduced fouling susceptibility.

Polyethylene and graphite are known for their low toxicity and their using at MED is not associated with great risk. However, a proper study of the possible degradation and subsequent leakage of any substances and their potential toxicity needs to be performed according standards to eliminate any health and environmental risks before these materials can be implemented for MED distillation.

## 4. Conclusions

HDPE-EG composites suitable for heat exchangers of multi-effect distillation have been prepared. 50 wt.% of EG increased thermal conductivity of HDPE to 2.18 W/m·K, corresponding to a 372% increase compared to neat HDPE. A series of plasma treatments were carried out to improve HDPE and HDPE/EG composites’ surface wettability and, therefore, reduce the scaling and fouling effects. The plasma treatment had remarkably enhanced the surface wettability with a maximum wettability achieved using 7 s of corona and 60 s of RF plasma treatment time. The plasma-treated HDPE/EG composite also exhibited remarkable wettability stability over time. A minor decrease in the surface free energy for RF and corona plasma of only 9% and 18% was observed, respectively). Moreover, the plasma-treated composite surface’s hydrophobic recovery was also less noticeable than that of plasma-treated neat HDPE that suffered a 14% and 20% decrease in the surface free energy for RF and corona plasma, respectively. 

Based on the investigation of the viscoelastic properties of prepared polymer composites, the mixtures below 30 wt.% of graphite in HDPE can be processed by the conventional extrusion process and shows typical rheological behavior for graphite-based polymer composites, while composites with EG loading above 30 wt.% require processing using an injection molding technique based on its complex viscosity profiles.

Moreover, the scaling measurements with CaSO_4_ and artificial seawater revealed that the plasma treatment of the HDPE-EG composite leads to a considerable reduction in the fouling tendency inducing superior fouling and scale resistance HDPPE-EG composites. The overall heat transfer coefficient of the plasma-treated HDPE-EG composite approaches that of stainless steel. Therefore, the HDPE-EG composite can compete favorably with the reference material, e.g., stainless steel, for heat exchanger applications for an MED desalination unit and other heat exchanger applications under similar conditions and environments.

## Figures and Tables

**Table 1:** Measured overall heat transfer coefficients for the investigated materials for various test conditions.

**Test Parameter**				
Re-hot water side	5840	5840	6600	6600
Re-cold water side	450	2700	450	2700
T hot in	70	70	80	80
T cold in	25	25	25	25
**Overall heat transfer coefficient U in W/m²**·**K**				
HDPE-EG-ut (s = 2 mm)	299.6	728.3	303.2	667.7
HDPE-EG-pt (s = 2 mm)	354.5	772.4	376.5	797.5
SS 1.4571 (s = 1 mm)	459.2	1241	385.4	1181

**Table 2:** Arithmetical mean heights of the investigated samples (surface roughness).

Material	S_a_ (Arithmetic Average Height)/µm
HDPE-EG-ut	0.698
HDPE-EG-pt	0.359
Stainless steel 1.4301	0.137
